# A Multicomponent Training Program Improves Physical Function and Quality of Life for a Mesenchymal Chondrosarcoma Survivor Subjected to Internal Hemipelvectomy: A Case Study

**DOI:** 10.3390/jcm14051541

**Published:** 2025-02-25

**Authors:** Lucía Guerrero Romero, Mar Cepero González, Francisco J. Rojas-Ruiz

**Affiliations:** 1 Centro de Entrenamiento Lucía Guerrero, 18004 Granada, Spain; lucia.guerreropt@gmail.com; 2 Department of Didactics of Musical, Plastic and Corporal Expression, University of Granada, 18071 Granada, Spain; mcepero@ugr.es; 3 Department of Physical Education and Sport, University of Granada, 18071 Granada, Spain

**Keywords:** physical activity, exercise modalities, cancer patients, quality of life

## Abstract

**Background/Objectives**: Overcoming an oncological process has a significant impact on lower-extremity sarcoma survivors’ quality of life, due to the deterioration in their physical and functional state. This study evaluated the effects of a six-month multicomponent physical training program on the physical function and quality of life of a mesenchymal chondrosarcoma survivor. **Methods**: The mesenchymal chondrosarcoma survivor was subjected to an internal hemipelvectomy including right hemipelvis plus femoral joint and resection of the right proximal femur followed by chemotherapy and radiotherapy according to the oncology protocol. The program was performed twice weekly and included resistance, cardiorespiratory, trunk control, and stability exercises. **Results:** Functional assessments revealed improvements in hand grip strength, walking speed, balance, and coordination. The Timed Up and Go Test showed a 50% reduction in completion time, reflecting better mobility and strength. Additionally, gait speed increased significantly, and balance trials indicated enhanced coordination. Quality of life evaluations indicated progress in physical health, psychological well-being, and environmental engagement. **Conclusions**: Taken together, this research emphasizes the importance of tailored exercise interventions for sarcoma survivors, particularly those with significant physical impairments. Such programs are vital complements to conventional rehabilitation strategies, fostering physical activity adapted to individual needs. By addressing the multifaceted challenges of survivorship, these interventions enhance functional capacity, reduce disability, and improve overall well-being. Therefore, this case study highlights the program’s effectiveness in managing post-treatment sequelae, opening a pathway to improved physical autonomy and quality of life.

## 1. Introduction

The incidence of cancer in the world is immense; as a matter of fact, it is the second cause of death after cardiovascular diseases. More than 19 million new cases of this disease worldwide were estimated to have appeared in 2020, causing 10 million deaths. It is estimated that these numbers will increase by almost 50% in 2040 [1]. Population growth and the increasing aging rates, as well as prevalent risk factors such as tobacco and alcohol use, physical inactivity, a sedentary lifestyle, and unhealthy diets, all contribute to the growing impact of cancer. Not only improvements in cancer therapy lead to an increase in the number of survivors, but also to improvements in early detection [2].

Sarcomas constitute a heterogeneous group of rare malignant neoplasms that arise from mesenchymal cells in soft tissues (about 84%, whose incidence increases with age too) and bones (more frequent in children and adolescents) [3]. They represent approximately 1% of all cancer types [4] and display very different development patterns at the time of diagnosis, treatment, and prognosis [5]. Around 60% of sarcomas in adults are located in the extremities [5] with frequent metastasis to the lungs. Depending on the pathology, location, and treatment of the tumor, they produce wide-ranging harmful effects on the survivors’ functionality and quality of life [5].

After osteosarcoma, chondrosarcoma is the most common of its kind, with an overall age-standardized incidence of 0.1 to 0.3 per 100,000 worldwide yearly. It accounts for 27% of all bone tumors according to the International Agency for Research on Cancer database (IARC) [6]. Moreover, chondrosarcoma is the most common sarcoma in adults between 20 and 60 years of age, even in the 40-year-old population. This type of cancer can manifest in any part of the body, although most arise in the extremities (45%) and the axial skeleton (31%). Within the appendicular skeleton, there is a strong predilection for the long bones of the lower extremity, whose most common location is the proximal femur [7].

Treatment for lower-extremity sarcomas may include a combination of surgical excision, chemotherapy, and/or radiotherapy, depending on the characteristics of the patient and the nature of the tumor [4,8]. The Sarcoma Unit of the Ruber International Hospital (Oncology Center in Madrid, Spain) has a highly qualified multidisciplinary team of specialists, dedicated to providing the diagnosis and treatment of musculoskeletal tumors or sarcomas, ensuring comprehensive and specialized care in this complex pathology.

All these treatments have a detrimental impact on the locomotor system of the patients suffering from musculoskeletal tumors which affect the pelvis and lower limbs [9]. Oftentimes, they cause muscle dysfunction due to decreased muscle strength and mass [9], and progressive worsening of gait and balance [10]. Indeed, the latter leads to reduced mobility, lower motor performance, lack of confidence and security in movement, loss of adaptation mechanisms to maintain the body in space, and increased risk of falls [10]. Hence, these alterations not only increase the possibility of fractures, disability, and loss of autonomy and independence but also promote physical inactivity and a sedentary lifestyle, accelerating the loss of functional capacity and the deterioration in quality of life [3,9].

Physical exercise intervention programs have been shown to be an essential complement to tackling the adverse side effects associated with various types of cancer [11,12,13]. However, there is still a lack of sufficient scientific evidence about the effects of exercise on survivors of mesenchymal chondrosarcoma, particularly in the stage following clinical treatment [11]. This research aimed to determine the effects and benefits of a multicomponent training program consisting of resistance, cardiorespiratory, trunk control, and stability exercises in improving the functional capacity and quality of life for a mesenchymal chondrosarcoma survivor. This program was carried out in a safe environment supervised by a physical sports educator. This allowed the individual to cope with the motor, cognitive, and emotional disorders that the disease (and post-treatment sequelae) entails, which remain a challenge for their recovery.

## 2. Case Presentation

A specific case is described below, as well as the effects of a 16-week multicomponent training program on physical function and quality of life.

### 2.1. Clinical Case

**Figure 1:**
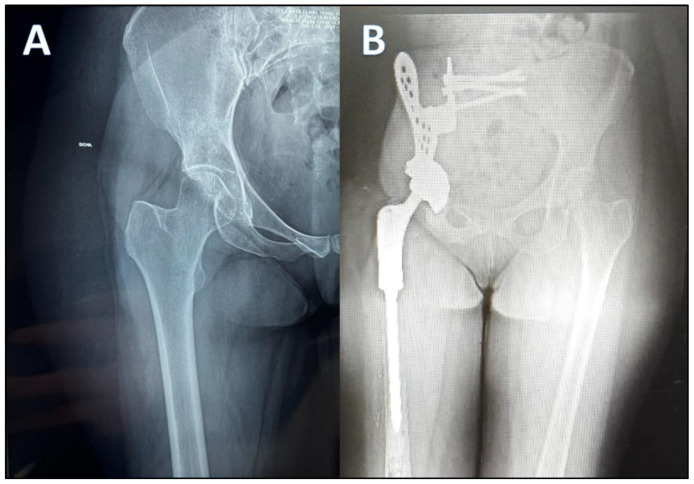
Aggressive tumor of apparent origin in the right acetabular region with poorly defined and permeable borders that gives rise to a large, solid, and heterogeneous soft tissue mass that infiltrates the right iliopsoas muscle. It presents intra-articular extension occupying the upper and anterior part of the joint, with reactive edema (**A**). Internal hemipelvectomy including right hemipelvis plus femoral joint and resection of the right proximal femur (**B**).

The participant is a 52-year-old woman residing in Granada (Spain), who survived a mesenchymal chondrosarcoma in the right hemipelvis diagnosed in 2015. Faced with an aggressive tumor of the right hemipelvis, of the mesenchymal chondrosarcoma type, the patient underwent surgery in September 2015 at the Ruber International Clinic (Madrid, Spain). Radiographic evaluation revealed an aggressive right iliac tumor with soft tissue extension infiltrating the iliac muscle and hip joint. The surgical intervention involved an internal hemipelvectomy and wide resection of zones 1 and 2 of the right hemipelvis. Extra-articular resection of the right proximal femur was performed in zones 1 and 2 of the right hemipelvis. Reconstruction with a custom-made prosthesis of the right hemipelvis (INPOL) and proximal femur (DePuy) was carried out. The hemipelvis prosthesis was osteosynthesized to the sacrum using cannulated screws and to the proximal femur with a double-mobility cup. Reconstruction of soft tissues used a Vertical Rectus Abdominis Myocutaneous (VRAM) flap (Figure 1). 

In November 2015, a new surgery was performed for acute periprosthetic infection by Enterococcus and Pseudomonas. This was controlled and chemotherapy and radiotherapy began according to the oncology protocol. In June 2016, she underwent surgery for a new hematogenous infection or exacerbation of the previous one, in the context of febrile neutropenia and radiotherapy. Coverage was performed with a pedicled flap of the homolateral vastus lateralis muscle. The patient had a right thoracotomy with resection of the metastatic nodule at the level of the right lower lobe (RLL) in 2017, and a year later, she suffered a Hoffa fracture of the internal condyle, thus requiring a new open reduction surgery and osteosynthesis with three screws. According to the medical report, the survivor had the following sequelae: resection of the hemipelvis and right hip; reconstruction with a tumor prosthesis, with arthrodesis of the sacrum and megaprosthesis in the hip, which causes great limitations in the mobility of the hip itself, the pelvis, and by extension, the entire lower body irreversibly. The patient is unable to perform activities that involve prolonged sitting, standing, or walking, or any activity that involves overloading the lower limbs. She endures chronic mechanical pain through the continuous intake of analgesics. She also suffers from lymphedema and chronic venous insufficiency. Improvement is not expected from these sequelae. In addition, she has an irreversible sciatic nerve injury, with a dropped foot that requires the use of an anti-equine orthopedic device producing chronic neuropathic pain. The third toe of the left foot is becoming stiff due to the nerve injury and poor support. Limitation of mobility in the right knee with a maximum flexion of 40 degrees is a sequela of surgery for the condylar fracture. She requires mobility in a wheelchair. Sometimes she can take a few steps aided by crutches.

The inclusion criteria adopted for the implementation of a multicomponent physical exercise program adapted to the patient’s characteristics and needs were the following: (1) female survivor of mesenchymal chondrosarcoma in the right hemipelvis; (2) having completed the early rehabilitation phase (24 h–3 months); (3) ability to walk independently with an assistive device (crutches); (4) score of at least 25 points or more on the Mini-Mental Test. The exclusion criteria were (1) inability to walk (considered as VM of 0 m·s^−1^); (2) presence of an acute or chronic disease that is not stabilized, or uncontrolled neurological conditions (epilepsy, loss of consciousness leading to falls), tumors, or metastases that make it difficult for her to participate in a supervised exercise program; and (3) any significant hearing or visual impairment. The participant provided her written informed consent and signed the data protection law.

#### Training Program

**Figure 2:**
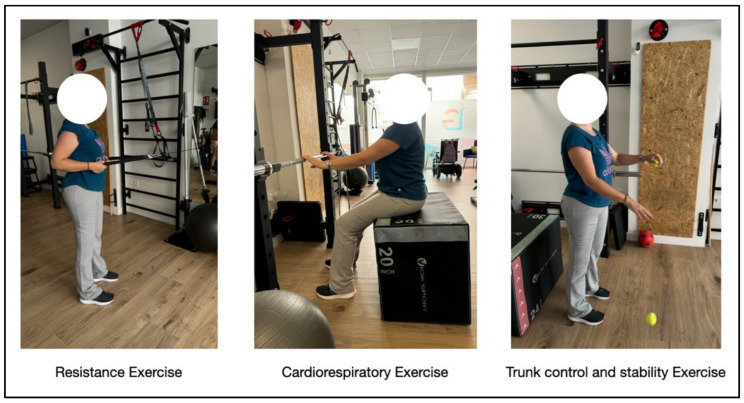
Examples of the exercise modalities included in the multicomponent training program.

The proposed multicomponent training program integrated the modalities of resistance, cardiorespiratory, and trunk control and stability training (Figure 2). It was customized and supervised by a physical sports educator (Col. 58601), with a duration of 6 months (November 2023–April 2024). Prior to its implementation, medical approval was received for the prescription of physical exercise. The program was then carefully planned (Table 1) through an initial interview and data collection in order to determine the initial physical condition and objectives, among other aspects. During the data collection process, the subject was given the “Physical Activity Aptitude Questionnaire (Par-Q)”, where she answered all questions negatively, as well as the “International Physical Activity Questionnaire (IPAQ)”, which placed her in the “low physical activity” category, since she did not have 5 or more days of moderate physical activity, did not walk at least 30 min per day, and sat for more than 6 h a day. These are useful tools to learn more about the levels of physical activity and sedentary behavior in the subject’s daily life. It is worth noting that, in this specific case, the results could have been influenced by physical symptoms and functional limitations, which made her aware of the effects and benefits that physical activity had on health and quality of life. Therefore, the recommendations were adapted to her abilities.

The training program consisted of a 24-week intervention (Table 2), carried out in two one-hour-long sessions per week. During the training sessions, heart rate, perceived exertion using the modified Borg scale, and pain and fatigue using the visual analog scale for pain and the fatigue assessment scale were monitored.

The resistance training proposed in the program (Table 3) was established with a low training volume (1–2 sets per exercise to progress to 3 sets), low intensity, an effort character of 4–6 repetitions/set performed on 12–15 RM (far from muscular failure and fatigue), and a moderate speed of execution. After familiarization and technical learning of the exercises in the first sessions of the program, the low loads were moved at the maximum intentional speed to generate neural adaptations and obtain benefits in the improvement of strength. Regarding the structure of the session, a global organization was chosen that involved upper- and lower-hemisphere exercises in the same session, which allowed for better time management and use, conditioned in part by the subject’s temporal availability. 

Physical exercises were selected for both large and small muscles (dorsi, pectoral, gluteal, quadriceps, hamstrings, deltoids, calves...), polyarticular (e.g., horizontal row or squat), and monoarticular (e.g., knee extension or elbow flexion) in order to ensure safety and effectiveness. The design of the stimuli took into account the most vulnerable joint nuclei affected by the surgery and the disease itself (muscle groups in the pelvis, hip, knee, and right ankle). Additionally, the muscle groups and motor gestures with greater prominence in the walking, sitting, and standing patterns were considered due to their importance in promoting the greatest possible autonomy in the performance of daily activities. The role played by the muscle groups of the shoulder girdle joints is also worth highlighting as they assist in moving with crutches, as well as daily gripping, pushing, and pulling activities.

Regarding cardiorespiratory training (Table 4), an initial volume of 10 min was proposed, progressing to 25 min in duration, with intensities of 50–75% of the heart rate reserve and a moderate subjective perception of effort (2–5 on the modified Borg scale). Fractional interval methodologies were mainly used for a better tolerance to effort, as well as continuous ones. The selection of exercises included cyclic (e.g., arm ergometer) and non-cyclic modalities (varied movements of greater complexity and motor richness).

Finally, training was designed so that exercise stimuli could specifically challenge trunk control and stability, along with other joint complexes such as the hip, knee, and ankle (Table 5). These challenges occur in the sagittal, frontal, and transverse planes and are primarily performed in two body positions: sitting and standing. The demands of internal stabilization were moderate, always avoiding loss of balance and falls, thus ensuring safe exercises, and a successful perception for the patient. In addition, the sensory organs also participated (visual, auditory, tactile stimuli). Dual tasks (motor stimulus and cognitive stimulus) were proposed to increase motor variability and motor exploration in space as well.

The above-described physical exercise program was complementary to the weekly compliance with the World Health Organization’s physical activity recommendations adapted to specific functional capacities and limitations, as well as other educational guidelines for a healthy lifestyle. Both the training program and the data collection and analysis were carried out at the facilities of “Lucía Guerrero Training Center” (Camino de Purchil, 5, CP 18,004 Granada, Spain).

### 2.2. Functional and Quality of Life Assessments

To assess functional capacity, prior to the start and after completion of the multicomponent training program, the following tests were performed: Hand Grip Strength (HGS) test with a hydraulic hand dynamometer (Baseline), Timed Up and Go (TUG) Test, 4 m Walking Speed Test, and March in Place Test (Optojump system). This is a brief explanation for each:

Firstly, hand grip strength as a “vital sign” is an essential metric to assess muscle function in a simple and objective way. It provides information on general physical capacity, is associated with the possible development of sarcopenia [14], and is essential as a biomarker of healthy aging and quality of life. Difficulties in carrying out daily activities such as getting up from a chair, climbing stairs, dressing or grooming, can be indicators of low HGS. In fact, low HGS values are an independent risk predictor for degenerative and inflammatory diseases, and in turn, diseases such as diabetes mellitus, arterial hypertension, or osteoarthritis increase the risk of low HGS [15].

Secondly, the TUG test, simple and economical, can be used to assess aspects related to the domains of gait, muscle strength and balance, which are in turn connected with a higher risk of falls. Indeed, aging, frailty, comorbidities, muscle dysfunction, balance impairment, and gait disorders can promote the risk of falls [16]. This is the reason why it was regarded as a fundamental test in this specific context.

In this regard, gait speed was also assessed, which is related to functional capacity and survival, a promising biomarker in the prevention of falls [17] and multiple pathologies including dementia and cognitive decline. The “4 m Walking Speed Test” was used, an inexpensive and easy-to-implement assessment tool [16]. However, gait speed may not be entirely informative about the way in which a person walks, although there are studies that point to the association between low gait speed and high stride-to-stride oscillations, i.e., high and low gait variability [18].

Finally, the “March in Place Test” was conducted with the Optojump system, which provides information regarding the precise comparison of the flight and contact times on the left and right sides to obtain a precise understanding of the coordination, balance, and synchronization of walking-in-the-same-place movements.

Regarding the evaluation of quality of life, before the beginning and after the training program, the “Quality of Life WHOQOL-BREF” questionnaire [19] was completed. This is an instrument derived from the WHOQOL-100 [20] in its short version, which comprises 26 questions, of which 24 refer to 4 dimensions: physical health, psychological health, social relationships, and environment.

## 3. Results

**Figure 3:**
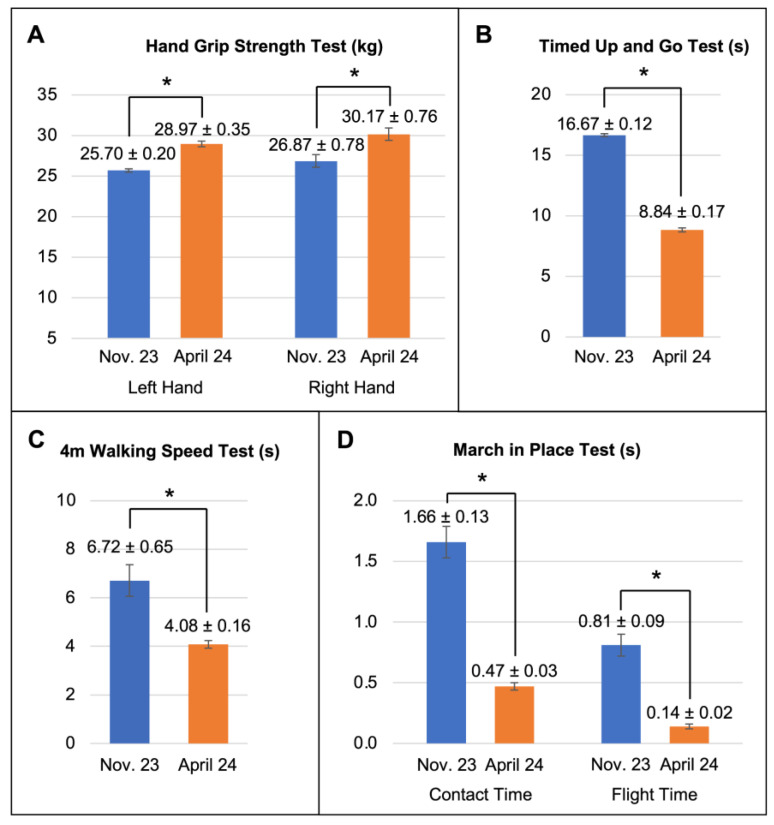
Results of the functional tests at the beginning and end of this study. (**A**) Hand Grip Strength Test (Baseline). (**B**) Timed Up and Go (TUG) Test. (**C**) The 4 m Walking Speed Test. (**D**) March in Place Test (Optojump) * denotes clear descriptive differences.

The results of the functional and quality of life tests were obtained at the beginning and after completing the training program (Figure 3 and Table 6). A significant increase in hand grip strength (or grip force) was detected after completing the physical exercise program, the right hand’s being greater than the left one’s (Figure 3A,B), likely due to the fact that the right is the dominant hand in most physical activities. Regarding the Timed Up and Go Test, there was a reduction of almost 50% in the time taken compared to the initial assessment, which seemed to indicate an improvement in walking, strength, and balance, as a result of the training program, and it was also correlated with the improvements found in the previous test.

Thirdly, the results obtained in the gait speed assessment (4 m walk test), as an objective measure of functional capacity and survival, showed a reduction of 2.64 s compared to the initial assessment (Figure 2). This reduction corresponded to a walking speed of 1.02 m per second in the final test, significantly lower than the value obtained in the initial test, 1.68 m per second.

Finally, we applied the March in Place Test based on two parameters, i.e., contact time and flight time (Figure 3). Both parameters showed lower values after the training program, which was a satisfactory result as a decrease in contact time promotes an increase in the number of steps, which in turn leads to a shorter flight time. In this way, the patient required less time to generate the force needed to increase the step frequency within a given time.

## 4. Discussion

The main purpose of this case study was to determine the effects and benefits of an individualized, supervised, multicomponent training program on physical function and health perception for a mesenchymal chondrosarcoma survivor. The results of the functional tests showed an improvement in functional capacity in terms of increased hand grip strength, increased walking speed due to improved times obtained in the “4 m Walking Speed Test” and “Time Up and Go (TUG) Test”, and possibly an improvement in balance, coordination, and synchronization of both lower limbs in the “March in Place Test”. It is true that, following this last test, a decrease in flight and contact times was observed, which could indicate a greater application of force in each push of the foot against the ground and its impulse and, therefore, greater step speed and number of contacts, and less permanence of each foot on the ground. It was suggested that the general increase in the subject’s strength levels as a result of carrying out the multicomponent training program had positively influenced the results achieved in the post-intervention “WHOQOL-BREF Quality of Life” questionnaire, reflecting a better general perception of health.

In relation to the publications reviewed to date on physical exercise in survivors of lower-extremity sarcoma, the scarce research found has focused on the implementation of exercise programs during treatment and immediately after surgery [21,22], as well as the evaluation of physical performance and quality of life around surgery and other treatments [3,8,9,11,23,24,25,26].

In fact, in reference centers, such as the Ruber Internacional Hospital (Madrid, Spain), they have a rehabilitation and physiotherapy team whose mission is to offer patients the opportunity to participate in prehabilitation through informative and educational sessions on exercise and healthy habits, as well as during and immediately after the treatments administered, in the hospital facilities to help with functional recovery and to be able to continue later at home or in specialized training centers supervised and directed by graduates in physical activity and sports sciences.

However, these studies mentioned above brought to light a lack of knowledge about physical exercise interventions once the treatment had finished, which also included various training modalities in which each of the dose variables was defined and controlled. Therefore, the authors consider this to be the first research study that attempts to design and implement a rigorous 24-week multicomponent training program which includes resistance, cardiorespiratory, and trunk control and stability training, with the aim of determining the effects and benefits of improving physical function and quality of life for a mesenchymal chondrosarcoma survivor.

Several measurement instruments are available for the assessment of functionality and quality of life; among them are the Musculoskeletal Tumor Society (MSTS) and the Toronto Extremity Salvage Score [9,27], both validated and used especially in survivors who have undergone limb salvage surgery. Nevertheless, scarce scientific evidence has been found regarding the development of specific tests that assess the gait, muscle function, and balance of lower-extremity sarcoma survivors. Specifically, the “Timed Up and Go Test” could be used in the objective evaluation of gait, muscle strength, balance, and risk of falls for this population, which encourages the authors to promote its practical application [9,10], and which is why it has been incorporated in the evaluation of the program.

Therefore, the authors of this research study used the Timed Up and Go Test and the HGS as they are objective and simple measures. The consequences of a low HGS on the general health of the population are recognized by multiple studies, and so are their links to sarcopenia [14,15]. Likewise, since there is no single assessment of gait, balance, and physical function, it was necessary to include the “4 m Walking Speed Test” because it has also been associated with functional capacity and general survival, especially in older adults [16], and the “March in Place Test” to complement information of interest regarding gait patterns, perhaps for this study’s subject particularly (or other cases of lower-extremity sarcoma), who has suffered from an illness and has undergone limb salvage surgery with subsequent notable physical and motor consequences.

For future research, the effects and benefits of a multicomponent training program should be analyzed in a larger sample of lower-extremity sarcoma survivors, in order to compare the results obtained and published to date. It is also hoped that the program design and its results will be useful and of practical application for health professionals, allowing for progress in research in this area and helping, through the best tool, physical exercise, to improve the health and well-being of lower-extremity sarcoma survivors.

## 5. Conclusions

One of the current problems in determining the effect and benefits of physical exercise interventions in lower-extremity sarcoma survivors is the heterogeneity of the training programs described in the literature, as well as the lack of a correct definition and control of the dose variables. To date, these have led to results that are difficult to interpret and/or of very low quality. The development and application of the multicomponent training program composed of several physical exercise modalities (resistance, cardiorespiratory, and trunk control and stability) have been shown to be an essential complement in improving functional capacity and quality of life necessary to tackle the challenges faced by a survivor of mesenchymal chondrosarcoma. A case series study should be conducted in the future taking into account that sarcoma patients could vary based on the level of resection, soft tissue involvement, staging, and type of reconstruction that has been used +/− flaps, and radiotherapy and/or chemotherapy administration. Herein lie the guidelines that will assist physical exercise professionals (graduates in physical activity and sports sciences) in the design and implementation of physical exercise interventions for adult sarcoma survivors, which promote physical activity and the improvement of health and quality of life.

## Figures and Tables

**Table 1:** Planning of the multicomponent training program.

Subject Level	Program Objectives	Spatial and Material Resources	Assessment Processes
Initial: untrained with little experience in regular physical exercise programs.	Functionality in daily living activities and quality of life.	Training center equipment (bench/box, rack, dumbbells, pulleys, elastic bands, medicine balls, bicycle, arm ergometer...).	1. Physical condition parameters (functional capacity): (A) Hand Grip Strength Test. (B) Timed Up and Go (TUG) Test. (C) The 4 m Walking Speed Test. (D) March in Place Test (Optojump system). 2. Quality of life: (A) WHOQOL-BREF.

**Table 2:** Periodization (time structures) of the training program composed of sessions with a multicomponent objective.

Mesocycles	M. 1	M. 2	M. 3	M. 4	M. 5	M. 6
Weekly frequency	2 d/w	2 d/w	2 d/w	2 d/w	2 d/w	2 d/w
Resistance training units	8	8	8	8	8	8
Cardiorespiratory training units	0	3	4	5	6	6
Trunk control and stability training units	8	6	5	4	4	4

**Table 3:** Resistance training programming and prescription. Example Mesocycle 1.

Frequency	Volume	Intensity	Recovery	Methodology	Exercises
2 d/w	-Training unit duration: 25–30′. -Exercises: 5. -Series: 2. -Repetitions: 6.	Low-effort character: 6 (12).	2′ inter-series.	Vertical progression.	-Assisted squat from box. -Horizontal pulley row. -Seated hip adduction with ball. -Inclined push-ups on bar. -Vertical traction with elastic band.

**Table 4:** Programming and prescription of cardiorespiratory training. Example Mesocycle 2.

Frequency	Volume	Intensity	Recovery	Methodology	Exercises
2 d/w	-Training unit duration: 10′ -Exercises: 2. -Series: 1. -Repetitions: 5 (30” each).	-50–60% HRR. -RPE: 2–3 (modified Borg)	1′ inter-repetitions. Ratio: 1:2.	Fractionation, interval.	Standing arm ergometer.

**Table 5:** Programming and prescription of trunk control and stability training. Example Mesocycle 3.

Frequency	Volume	Intensity	Recovery	Methodology	Exercises
2 d/w	-Training unit duration: 12′ -Exercises: 3. -Series: 2. -Repetitions: 6–10	Challenge/disruption for the trunk structures.	2′ inter-series.	Vertical progression.	-Alternately bounce 2 tennis balls with eyes closed while standing. -Apply manual resistance to hands in multiple directions while sitting. -Stand with horizontal shoulder adduction, rotate the cervical spine from one side to the other when receiving an auditory stimulus (clap).

**Table 6:** Results of the WHOQOL-BREF Quality of Life questionnaire obtained at the beginning and end of the training program.

Dimensions	November 2023 (Pre-Intervention)	April 2024 (Post-Intervention)
Physical health	10.3/20	10.9/20
Psychological health	12/20	13.3/20
Social relations	14.7/20	14.7/20
Environment	15.5/20	16/20
General perception of quality of life	4/5	4/5
General perception of health	3/5	4/5

## Data Availability

Centro de Entrenamiento Lucía Guerrero, 18004 Granada, Spain.
